# Electrospinning preparation of g-C_3_N_4_/Nb_2_O_5_ nanofibers heterojunction for enhanced photocatalytic degradation of organic pollutants in water

**DOI:** 10.1038/s41598-021-02161-x

**Published:** 2021-11-25

**Authors:** Lu Wang, Ya Li, Pingfang Han

**Affiliations:** 1 College of Biotechnology and Pharmaceutical Engineering, Nanjing Tech University, Nanjing, 211816 China; 2 Nantong Vocational University, Nantong, 226007 China; 3 Nantong College of Science and Technology, Nantong, 226007 China

**Keywords:** Environmental sciences, Nanoscience and technology

## Abstract

In this study, graphitic carbon nitride (g-C_3_N_4_) and niobium pentoxide nanofibers (Nb_2_O_5_ NFs) heterojunction was prepared by means of a direct electrospinning approach combined with calcination process. The characterizations confirmed a well-defined morphology of the g-C_3_N_4_/Nb_2_O_5_ heterojunction in which Nb_2_O_5_ NFs were tightly attached onto g-C_3_N_4_ nanosheets. Compared to pure g-C_3_N_4_ and Nb_2_O_5_ NFs, the as-prepared g-C_3_N_4_/Nb_2_O_5_ heterojunction exhibited remarkably enhanced photocatalytic activity for degradation of rhodamine B and phenol under visible light irradiation. The enhanced catalytic activity was attributed predominantly to the synergistic effect between g-C_3_N_4_ sheets and Nb_2_O_5_ NFs, which promoted the transferring of carriers and prohibited their recombination, confirmed by the measurement of transient photocurrent responses and photoluminescence spectra. In addition, the active species trapping experiments indicated that superoxide radical anion (·O_2_^–^) and hole (h^+^) were the major active species contributing to the photocatalytic process. With its high efficacy and ease of preparation, g-C_3_N_4_/Nb_2_O_5_ heterojunction has great potentials for applications in treatment of organic pollutants and conversion of solar energy.

## Introduction

In recent years, water pollution caused by textile dyes and other organic pollutants has made serious damage to the ecosystem and human health as they are toxic, mutagenic, and mostly non biodegradable^1,2^. For the sustainable development of human being, there is urgent demand to remove water contamination. Traditionally, physical, chemical, and biological wastewater treatment processes are used but generally have several disadvantage such as high cost, low degradation efficiency, etc.^3^. Recently, as an ideal “green strategy” to deal with increasing environmental issues, metal oxide semiconductor based photocatalysis has drawn great attention because of their versatile properties^4,5^. In photocatalysis process, when the semiconductors are illuminated by photons with energy higher than their band gap, active charges are generated to cause photocatalytic reactions toward pollutant degradation^6^. Nowadays, semiconductor materials such as TiO_2_, ZnO, Nb_2_O_5_, CeO_2_, BiOI, graphene, g-C_3_N_4_ and their heterojunction composites like Nb_2_O_5_/TiO_2_, BiOI/TiO_2_, g-C_3_N_4_/TiO_2_, Nb_2_O_5_/ZnO and CeO_2_/Nb_2_O_5_ have been used to overcome the water pollution issues^7–20^. Among these semiconductor materials, Nb_2_O_5_, a promising traditional semiconductor material with a band gap of ca. 3.2 eV, has been widely used in a variety of fields, for instance, electrode materials, catalysis, photodecomposition of water and especially photodedegradation of harmful organic pollutants in water, due to its outstanding advantages of thermodynamic stability, nontoxicity and relatively high photocatalytic activity^21^. However, the rapid recombination of photogenerated charges hindered the practical application of the pure Nb_2_O_5_, similar to other traditional semiconductor photocatalysts^22,23^. With the purpose of facilitating the separation of photoinduced charge, novel Nb_2_O_5_-based composites which are suitable for catalysis of pollutant degradation should be constructed. Consequently, researches found that Nb_2_O_5_-based heterojunctions with other materials, such as metal (Ag, Au, Pt, etc.), metal oxide (TiO_2_, NiO, Ag_2_O, Fe_2_O_3_, etc.) and graphene is a prominent method^24–30^. Particularly, Nb_2_O_5_ heterojunctions coupled with visible-light-responsive semiconductors are recognized as the most effective photocatalysts for wastewater treatment because of the internal electric field, which can suppress the recombination of photogenerated charge and effectively improve mutual transfer of photogenerated charge in the heterojunctions, thus ultimately enhance the photocatalytic activity. In addition, visible-light-responsive semiconductor coupling Nb_2_O_5_ can also improve the light absorption capacity to a significant extent^31^.

g-C_3_N_4_, a two dimensional polymeric semiconductor, has attracted much attention in recently years due to its unique delocalized conjugated π structure formed by sp^2^ hybridization of C and N atoms which offers a rapid photoinduced charge separation in the electron transfer process^32^. In particular, g-C_3_N_4_ can activate molecular oxygen and produce superoxide radicals, which effectively enhances the activities for photocatalytic reactions. Furthermore, g-C_3_N_4_ shows massive prospects for application of photocatalytic degradation due to its advantages of narrow band gap, low cost, eco-friendliness and excellent optical and thermal properties^33,34^. Unfortunately, the photocatalytic activity of pure g-C_3_N_4_ is also usually restricted because of the fast recombination of photogenerated electron/hole pairs. In order to improve its efficiency in photocatalytic processes, strategies such as coupling g-C_3_N_4_ with TiO_2_, CeO_2_, BiVO_4_ have been proposed, which can not only effectively reduce the photoinduced electron–hole recombination rate, but also form intermediate energy levels in the forbidden band of metal oxide owing to the matching band structure of g-C_3_N_4_ and metal oxide^35–37^. So far, many g-C_3_N_4_/Nb_2_O_5_ heterojunction photocatalysts have been successfully prepared to enhance photocatalytic activity for pollutants degradation due to the well match of band gap edges between Nb_2_O_5_ and g-C_3_N_4_, which facilitates the charge carrier separation and thus improves the photocatalytic performance. Carvalho et al. reported that g-C_3_N_4_/Nb_2_O_5_ heterojunction photocatalysts, assembled as Nb_2_O_5_ nanoparticles decorating the g-C_3_N_4_ surface via hydrothermal process, exhibited remarkable enhanced photocatalytic activity in the degradation of methylene blue and rhodamine B dyes^38^. Silva et al. reported that g-C_3_N_4_/Nb_2_O_5_ heterostructures, assembled as Nb_2_O_5_ nanospheres decorating the g-C_3_N_4_ surface by a sonochemical method, showed high activity for dye and drug pollutants degradation under visible light irradiation^39^. Hong et al. reported Nb_2_O_5_/g-C_3_N_4_ heterojunctions prepared by a simple heating method showed significantly enhanced photocatalytic activity in the degradation of tetracycline hydrochloride^40^. As shown in these reports, the g-C_3_N_4_/Nb_2_O_5_ heterojunction had excellent photocatalytic performance and remarkable optoelectronic characteristics for degrading organic pollutants in wastewater compared with individual g-C_3_N_4_ and pure Nb_2_O_5_. Nevertheless, most of these studies were based on powder-form Nb_2_O_5_, which was prepared by complicated means such as solvothermal method, chemical precipitation method, et al. These conventional preparation methods may lead to agglomeration of Nb_2_O_5_ nanoparticles and then reduce the photocatalytic activity. Hence, it is of great interest to develop a facile and practical method for effective preparation of the evenly nanostructured g-C_3_N_4_/Nb_2_O_5_ heterojunction with large specific surface area and improved photocatalytic activity.

To date, one-dimensional semiconductor metal oxide nanofibers fabricated through electrospinning have been greatly attractive due to the advantages of high surface areas and large surface-to-volume ratio, which can provide quick charge transfer channels and more active sites^41–44^. More significantly, the electrospun nanofibers with a rather high surface area will be optimal carriers for fabricating heterojunction photocatalysts and then provide more active reaction sites for interaction with pollutants^45^. Meanwhile, the electrospun nanofibers with nonwoven web structure results in an easy separation and recovery from fluid in photocatalytic process^46,47^. On the basis of above, constructing heterojunctions of electrospun Nb_2_O_5_ nanofibers coupled with g-C_3_N_4_ would be expected as promising composite photocatalyst for practical applications.

In the present work, we developed a g-C_3_N_4_/Nb_2_O_5_ nanofibers heterojunction via a simple electrospinning technique, which exhibited a photocatalytic activity superior to the pure g-C_3_N_4_ and electrospun Nb_2_O_5_ NFs. Meanwhile, the as-prepared heterojunction could be recovered easily by filtration without reducing the photocatalytic activity. Moreover, a possible degradation mechanism is also proposed based on the detailed structural analysis of the heterojunction.

## Experimental

### Synthesis of g-C_3_N_4_/Nb_2_O_5_ heterojunction

In a typical procedure, g-C_3_N_4_/Nb_2_O_5_ heterojunction was synthesized by electrospinning. First, 10 g of CH_6_ClN_3_ was heated in an open crucible in static air at a heating rate of 10 °C/min to 600 °C and kept at that temperature for 4 h. The product was collected and ground into powder in an agate mortar to obtain the g-C_3_N_4_. Subsequently, 0.25 g of NbCl_5_ and 0.025 g of g-C_3_N_4_ were added into 2.65 mL of *N*,*N*-dimethylformamide (DMF) and ultrasonicated for 1 h. Then 0.35 g of polyvinylpyrrolidone (PVP) was dissolved in the mixture. After magnetic stirring for 8 h, the precursor solution of g-C_3_N_4_/NbCl_5_/PVP composite was afforded and transferred into a 5 mL plastic syringe with a 25 gauge stainless steel needle for electrospinning. In this electrospinning experiment, the collector was positioned 20 cm away from the tip of needle and the applied direct voltage between the collector and the needle tip was ~ 18 kV, the precursor solution flow rate was 0.25 mL/h. Then, the collected precursor nanofibers were calcined in muffle furnace at 600 °C for 1 h in air with a heating rate of 10 °C/min to obtain g-C_3_N_4_/Nb_2_O_5_ heterojunction. For comparison, pure Nb_2_O_5_ nanofibers was prepared under the same condition without adding g-C_3_N_4_.

### Characterizations and photocatalytic experiment

Supporting Materials showed their details.

## Results and discussion

### Characterization

**Figure 1:**
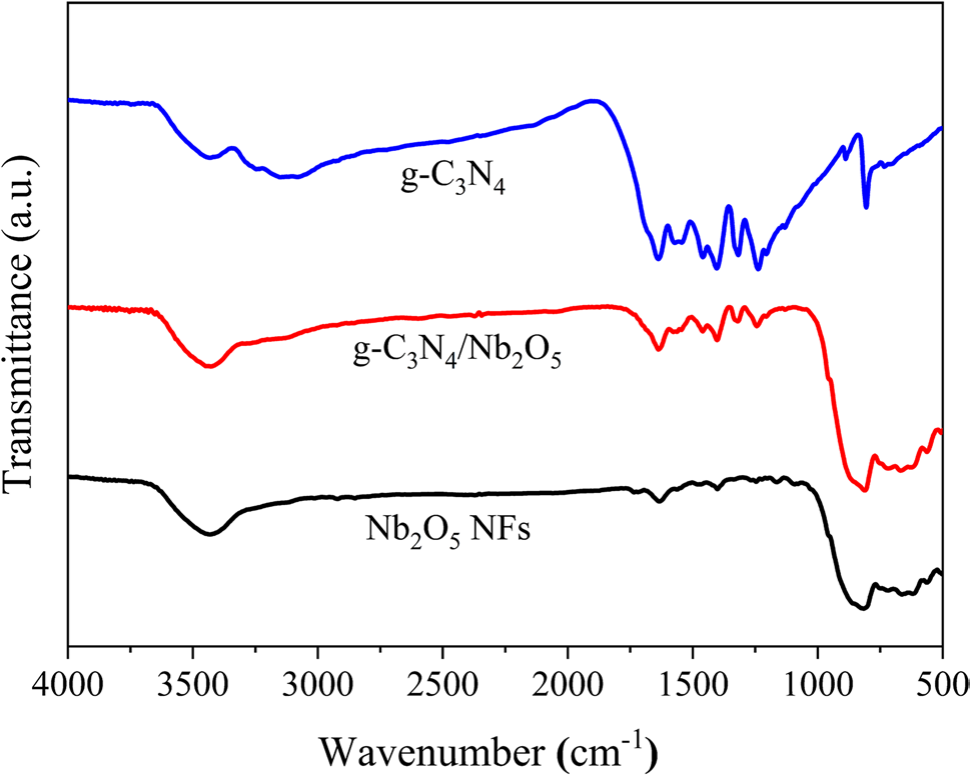
FTIR spectrum of g-C_3_N_4_, Nb_2_O_5_ NFs and g-C_3_N_4_/Nb_2_O_5_ heterojunction.

The Fourier Transform infrared (FTIR) spectra of as prepared samples were shown in Fig. 1. In the FTIR spectrum of Nb_2_O_5_ NFs, the peaks at 827 cm^−1^ and 665 cm^−1^ were assigned to Nb–O–Nb and Nb=O bonds of Nb_2_O_5_, respectively^48^. For g-C_3_N_4_, the broad adsorption band centered at 3188 cm^−1^ and the peak at 1637 cm^−1^ belong to the stretching vibration mode of terminal NH groups and the C=N stretching vibration modes, respectively. The peaks around at 1411 cm^−1^, 1317 cm^−1^ and 1238 cm^−1^ were attributed to aromatic C–N stretching. The unique absorption peak at approximately 806 cm^−1^ was related to the s-triazine ring modes^49^. As expected, all of the major characteristic absorption peaks of g-C_3_N_4_ and Nb_2_O_5_ were present in the spectrum of g-C_3_N_4_/Nb_2_O_5_ heterojunction, suggesting that composite samples contained g-C_3_N_4_.

**Figure 2:**
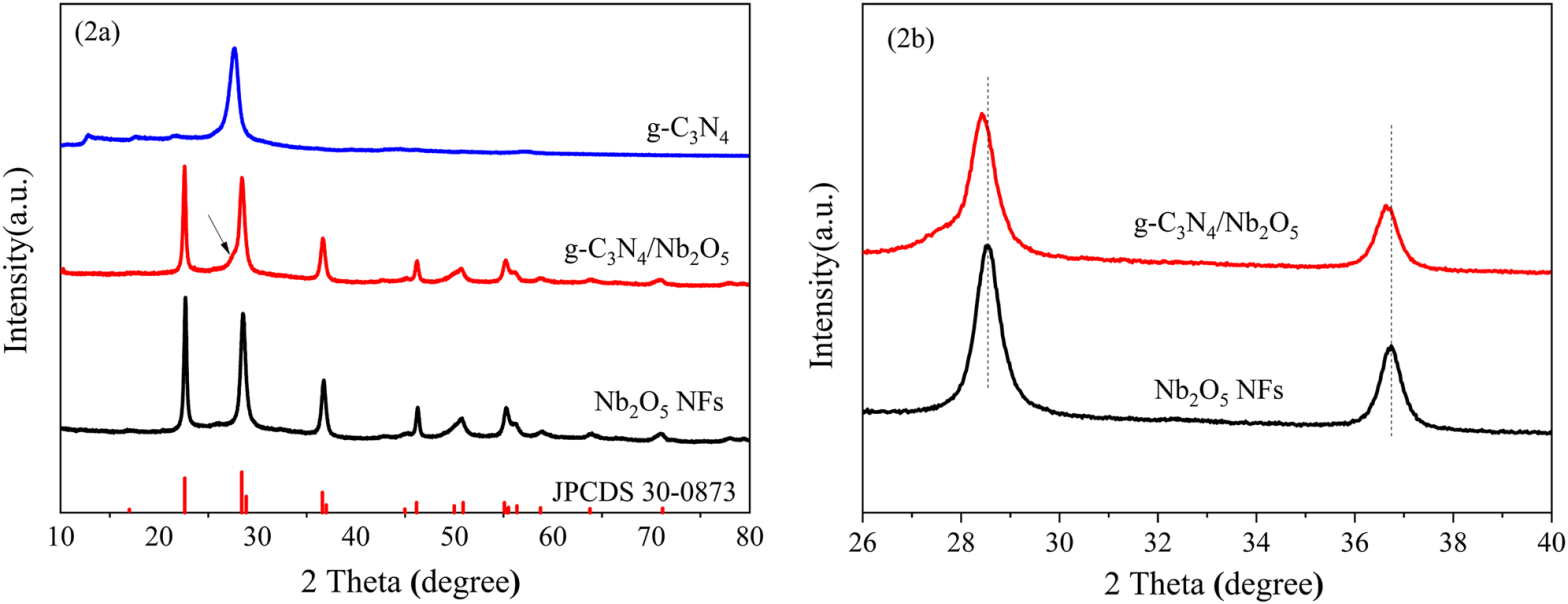
(**a**) XRD patterns of g-C_3_N_4_, Nb_2_O_5_ NFs and g-C_3_N_4_/Nb_2_O_5_ heterojunction and (**b**) enlarge of 2 theta scale at 26°–40° region of Nb_2_O_5_ NFs and g-C_3_N_4_/Nb_2_O_5_ heterojunction.

Figure 2 depicted the X-ray diffraction (XRD) patterns of g-C_3_N_4_, Nb_2_O_5_ NFs and g-C_3_N_4_/Nb_2_O_5_ heterojunction. As shown in Fig. 2a, the quintessential characteristic diffraction peaks of pure g-C_3_N_4_ at 13.4° and 27.6° associated to the (100) and (002) planes of the graphite-like structure of C_3_N_4,_ respectively^50^. The XRD pattern revealed that the diffraction peaks of Nb_2_O_5_ NFs correspond to the orthorhombic phase (standard JCPDS card 30-0873). It is noticeable that the synthesized g-C_3_N_4_/Nb_2_O_5_ heterojunction exhibited similar pattern to Nb_2_O_5_ NFs. The characteristic peak of g-C_3_N_4_ (002) closed to orthorhombic (180) peak was not extrusive in the pattern of g-C_3_N_4_/Nb_2_O_5_ heterojunction owing to the comparatively low dosage of g-C_3_N_4_. Moreover, compared with Nb_2_O_5_ NFs, the diffraction peaks of Nb_2_O_5_ in the heterojunction became weaker and shifted slightly to a smaller value diffraction angle (Fig. 2b) with the addition of g-C_3_N_4_, which may be caused by the interaction between g-C_3_N_4_ and Nb_2_O_5_ in the heterojunction. Similar phenomenon was also reported in an earlier literature^51^.

Fig. S1 showed the thermogravimetric analysis (TGA) curves of g-C_3_N_4_ and g-C_3_N_4_/Nb_2_O_5_ heterojunction. The thermal profile of g-C_3_N_4_ indicated that the as-prepared material was stable in air flow below 600 °C, and heating to 700 °C resulted in no residue of the material being observable^52^. The weight of the g-C_3_N_4_/Nb_2_O_5_ heterojunction decreased rapidly in the temperature range 600–700 °C, owing to the demoposition of g-C_3_N_4_ occurred in this temperature range. Hence, the g-C_3_N_4_ content for the g-C_3_N_4_/Nb_2_O_5_ heterostructure could be estimated to be 9.2 wt% neglecting the amount of surface-bound water.

**Figure 3:**
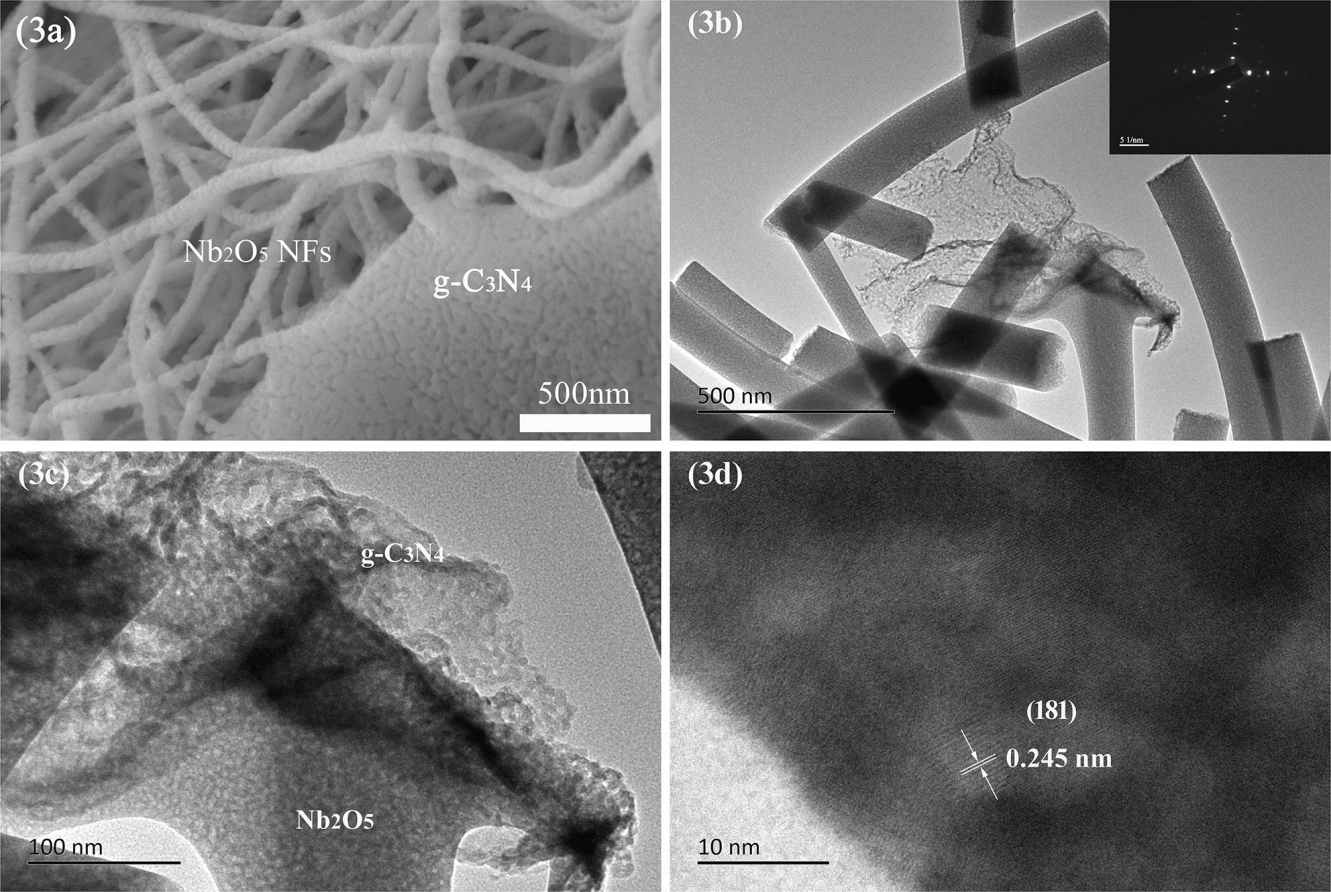
(**a**) SEM, (**b**,**c**) TEM and (**d**) high resolution TEM images of g-C_3_N_4_/Nb_2_O_5_ heterojunction. The inset of (**b**) is SAED pattern of g-C_3_N_4_/Nb_2_O_5_ heterojunction.

The morphology of g-C_3_N_4_, Nb_2_O_5_ NFs and g-C_3_N_4_/Nb_2_O_5_ heterojunction were observed by scanning electron microscopy (SEM) and transmission electron microscope (TEM). It was found that the Nb_2_O_5_ NFs represent 1D nanofiber morphology, which had an average diameter of 150 nm with random orientation (Fig. S2a). The g-C_3_N_4_ sample displayed an irregular sheet like structure (Fig. S2b). It was perceptible on the SEM image of g-C_3_N_4_/Nb_2_O_5_ heterojunction (Fig. 3a) that two semiconductors, Nb_2_O_5_ nanofibers and g-C_3_N_4_, directly and sufficiently contacted through mixed eletrospinning process and calcination. Such line/area contact increased porosity-related characteristics of the heterojunction and resulted in less agglomeration. In particular, such heterojunction formed by nanofiber and nanosheet can enhance the transport of charges and reduce the recombination probability of photoexcited charge carriers, ultimately further improve the photodegradation efficiency^53^. TEM image (Fig. 3b) further revealed that the g-C_3_N_4_/Nb_2_O_5_ heterojunction was composed of homogeneous, long and narrow Nb_2_O_5_ NFs that were in direct contact with g-C_3_N_4_ nanosheets. In addition, the selected area electron diffraction (SAED) pattern (inset of Fig. 3b) depicted broad and strong spots that was assigned to the essentially 1D characteristic of the long nanofibers, indicating the highly single crystallinity of the orthorhombic-phase Nb_2_O_5_^54,55^, which was in accordance with the XRD analysis results of Nb_2_O_5_. No diffraction spots/rings of g-C_3_N_4_ were detected in the SAED spectrum, indicating that g-C_3_N_4_ was amorphous in the g-C_3_N_4_/Nb_2_O_5_ heterojunction^56^. Figure 3c exhibited that Nb_2_O_5_ NFs with a distribution of diameters ~ 150 nm were attached onto the surfaces of g-C_3_N_4_ nanosheets, forming g-C_3_N_4_/Nb_2_O_5_ heterojunction structure. Moreover, the high resolution TEM image (Fig. 3d) revealed the lattice fringe with d-spacing of approximately 0.245 nm, which was in agreement with the (181) spacing of orthorhombic Nb_2_O_5_^57^. With these, a heterojunction formation between Nb_2_O_5_ and g-C_3_N_4_ was confirmed. This heterojunction would promote charge transfer between Nb_2_O_5_ and g-C_3_N_4_ and separation of photogenerated electron–hole pairs, both of which would enhance the photocatalytic activity.

**Figure 4:**
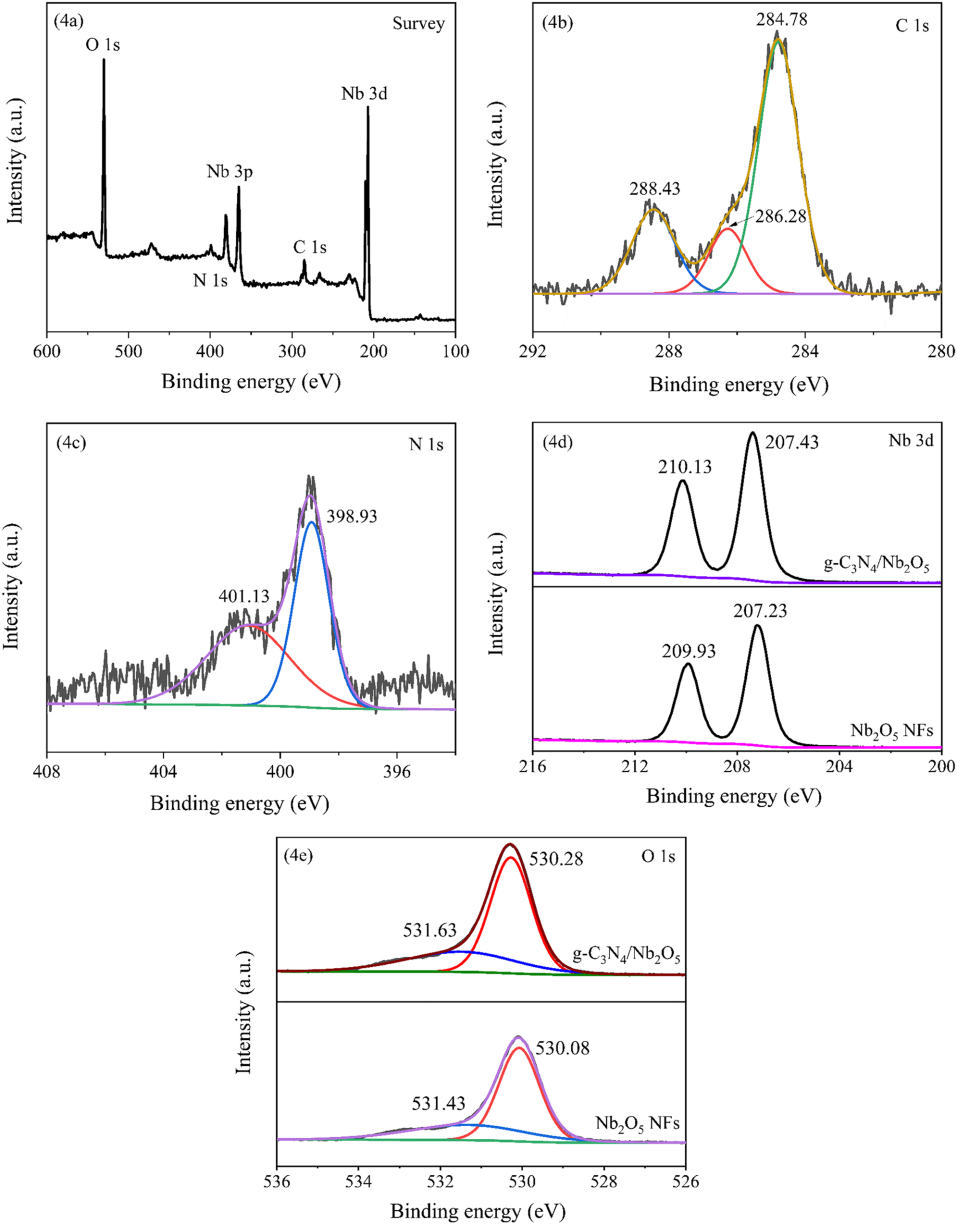
(**a**) XPS survey spectrum and high resolution XPS spectra of (**b**) C 1s, (**c**) N 1s, (**d**) Nb 3d and (**e**) O 1s in g-C_3_N_4_/Nb_2_O_5_ heterojunction.

The X-ray photoelectron spectroscopy (XPS) survey spectrum and high resolution spectrum of g-C_3_N_4_/Nb_2_O_5_ heterojunction were performed to illuminate the surface composition and the chemical environment. As shown in Fig. 4a, the survey XPS spectrum exhibited that the g-C_3_N_4_/Nb_2_O_5_ heterojunction not only contained Nb, O elements related to the Nb_2_O_5_ phase, but also contained C, N elements related to the g-C_3_N_4_ phase. Correspondingly, the C 1 s high-resolution spectra (Fig. 4b) was divided into three fitted peaks at 284.78 eV, 286.28 eV and 288.43 eV. The peak at 284.78 eV corresponded to sp^2^-hybridized carbon atoms of the carbon standard used to calibrate the binding energies. The peaks at 286.28 eV and 288.43 eV was attributed to the C–N–C and N–C=N backbones coordination in the triazine rings of g-C_3_N_4_, respectively^58^. The N 1s high-resolution spectra (Fig. 4c) was deconvoluted into two fitted peaks at 398.93 eV and 401.13 eV, which could be assigned to N sp^2^-bonded to C (N-sp^2^C) and tertiary nitrogen groups (N-(C)_3_) of g-C_3_N_4_, respectively^59^. The Nb 3d spectrum (Fig. 4d) exhibited two significant peaks at around 207.43 and 210.13 eV, corresponding to 3d_5/2_ and 3d_3/2_ states of Nb^5+^, respectively^60^. The O 1s XPS spectrum (Fig. 4e) could be fitted to two peaks centered at around 530.28 and 531.63 eV, which were assigned to the Nb–O bond and adsorbed oxygen, respectively. Notably, compared with those of pure Nb_2_O_5_ NFs, the Nb 3d and O 1s peaks of g-C_3_N_4_/Nb_2_O_5_ heterojunction shifted slightly to higher energies, evidencing the intense interaction between the Nb_2_O_5_ and g-C_3_N_4_ that resulted from the formation of effective heterojunction. These phenomena are in good agreement with previous report^53^.

**Figure 5:**
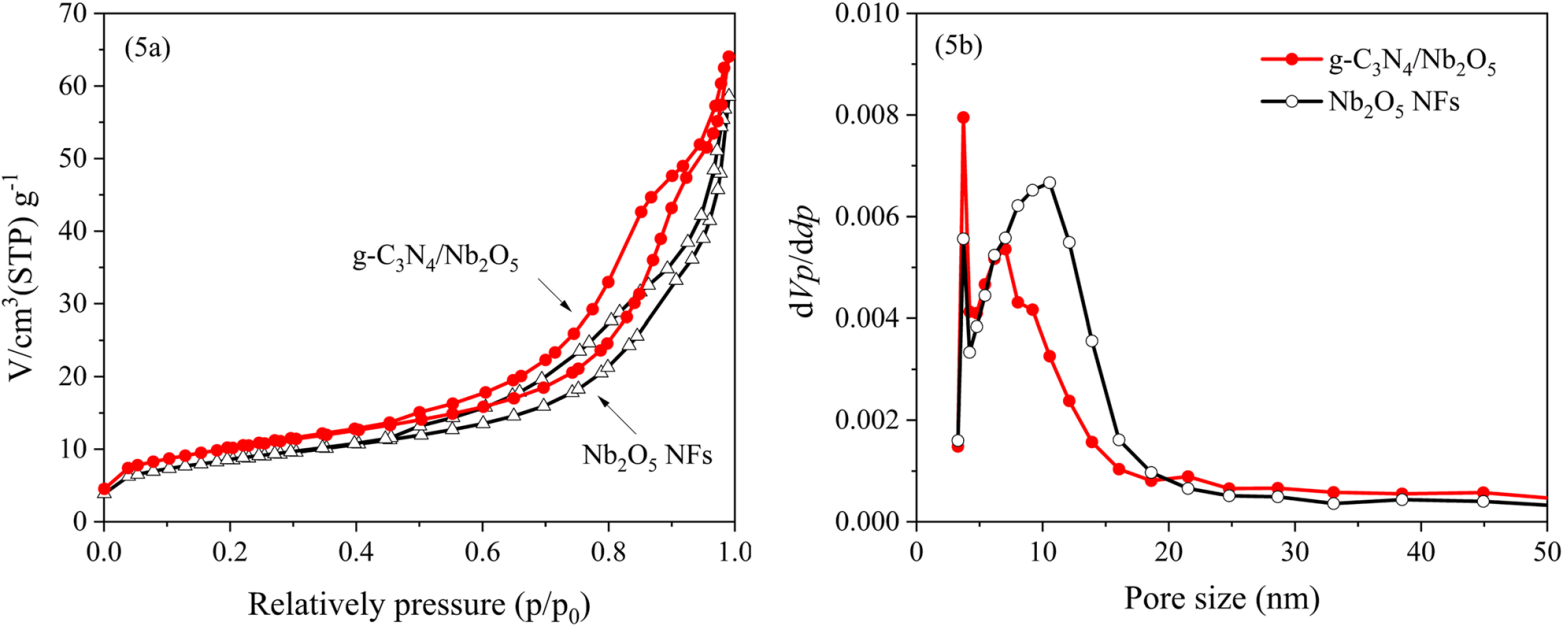
(**a**) N_2_ sorption isotherm and (**b**) pore size distribution of Nb_2_O_5_ NFs and g-C_3_N_4_/Nb_2_O_5_ heterojunction.

Figure 5 showed N_2_ absorption–desorption isotherms and pore size distribution curves of Nb_2_O_5_ NFs and g-C_3_N_4_/Nb_2_O_5_ heterojunction. As illustrated in Fig. 5a, both the isotherms belonged to type IV isotherm possessed obvious type H_3_ hysteresis loop at relative higher *p*/*p*_0_, suggesting the existence of slit-shaped pores due to the aggregation of nanoparticles^61^. The BET specific surface areas of Nb_2_O_5_ NFs and g-C_3_N_4_/Nb_2_O_5_ heterojunction are 30.09 m^2^/g and 36.18 m^2^/g, respectively. In addition, Fig. 5b displays the pore-size distributions of Nb_2_O_5_ NFs and g-C_3_N_4_/Nb_2_O_5_ heterojunction estimated with the BJH method. As seen from the spectra, two characteristic diameters of mesopores (2–50 nm) are located primarily at 3.7 and 10.6 nm for pure Nb_2_O_5_ NFs, 3.7 and 7.1 nm for g-C_3_N_4_/Nb_2_O_5_ heterojunction, respectively. The increased BET specific surface area and decreased pore size were likely caused by the incorporation of g-C_3_N_4_, which was fundamental in enhancing the photodegradation activity of electrospun g-C_3_N_4_/Nb_2_O_5_ heterojunction.

**Figure 6:**
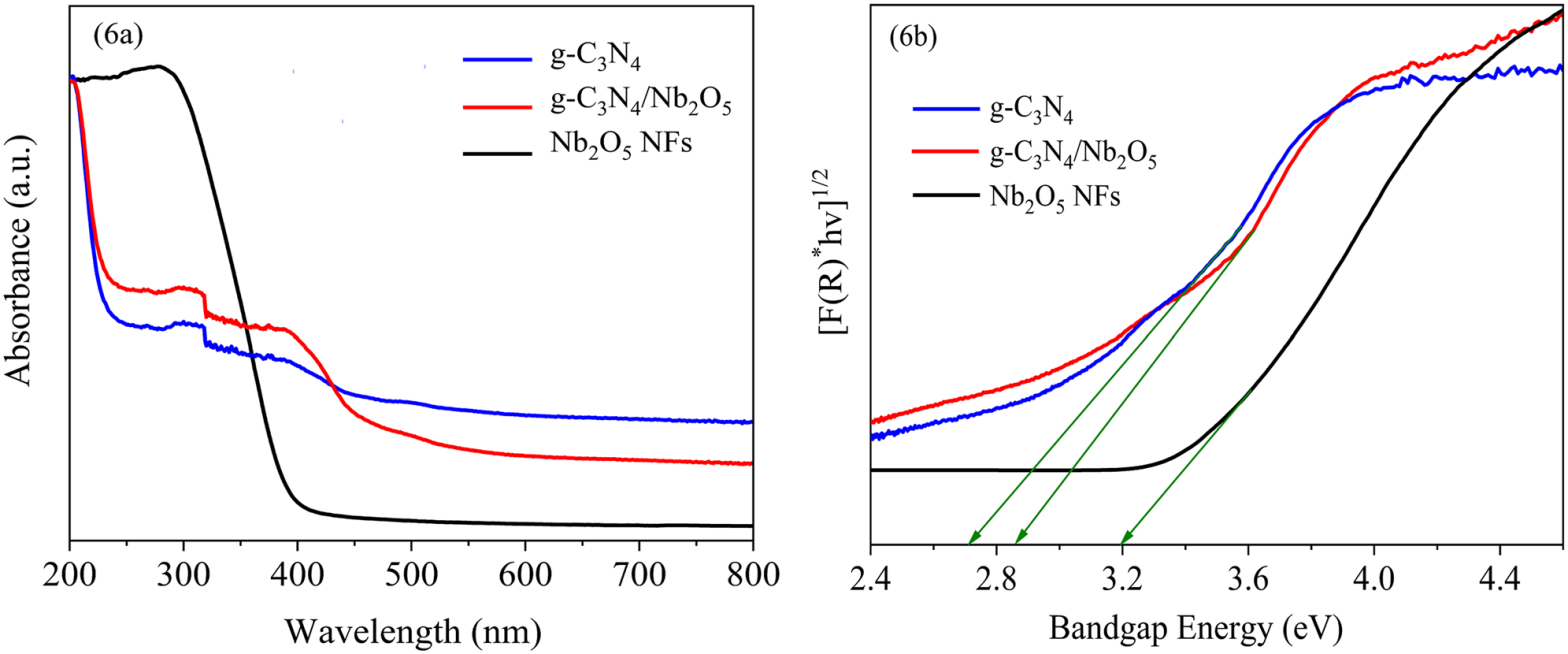
(**a**) UV–vis diffused reflectance spectra and (**b**) Tauc plot graph of g-C_3_N_4_, Nb_2_O_5_ NFs and g-C_3_N_4_/Nb_2_O_5_ heterojunction.

The UV–Vis absorption spectra of as-prepared samples were depicted in Fig. 6a. It is obvious that the g-C_3_N_4_ has absorption in the visible region, which is in good agreement with the narrow band gap. However, the Nb_2_O_5_ NFs only absorbs UV light, consistent with wide band gap. Compared to Nb_2_O_5_ NFs, the absorption edge of g-C_3_N_4_/Nb_2_O_5_ heterojunction shifts to the visible light region with the absorbance edge between those of Nb_2_O_5_ NFs and g-C_3_N_4_. It can be concluded that the combination of g-C_3_N_4_ and Nb_2_O_5_ may enhance the visible light absorption of the sample and thus improve their catalytic activity in the visible region^35^. In addition, the bandgap energy of samples were obtained from Tauc plot by extrapolation to the photon energy-axis. As illustrated in Fig. 6b, the band gap values of the g-C_3_N_4_ and Nb_2_O_5_ NFs were estimated to be approximately 2.7 and 3.2 eV, respectively, consistent with those described for these phases in the literature^31,62^. In addition, the band gap of g-C_3_N_4_/Nb_2_O_5_ heterojunction was found to be 2.84 eV. This suggested that the synthetized g-C_3_N_4_/Nb_2_O_5_ heterojunction had smaller band gap than that of pure Nb_2_O_5_ NFs, which was the precondition of effective photocatalytic activity in visible region. Besides, Mott–Schottky plots (Fig. S3) of the Nb_2_O_5_ NFs and g-C_3_N_4_ were collected to define their conduction band potential (E_CB_). The derived E_CB_ of Nb_2_O_5_ NFs and g-C_3_N_4_ were estimated to be − 0.59 and − 0.98 eV, respectively^63^. Thus, the valence band potential (E_VB_) of them were calculated to be 2.61 and 1.72 eV, respectively.

### Photocatalysis

**Figure 7:**
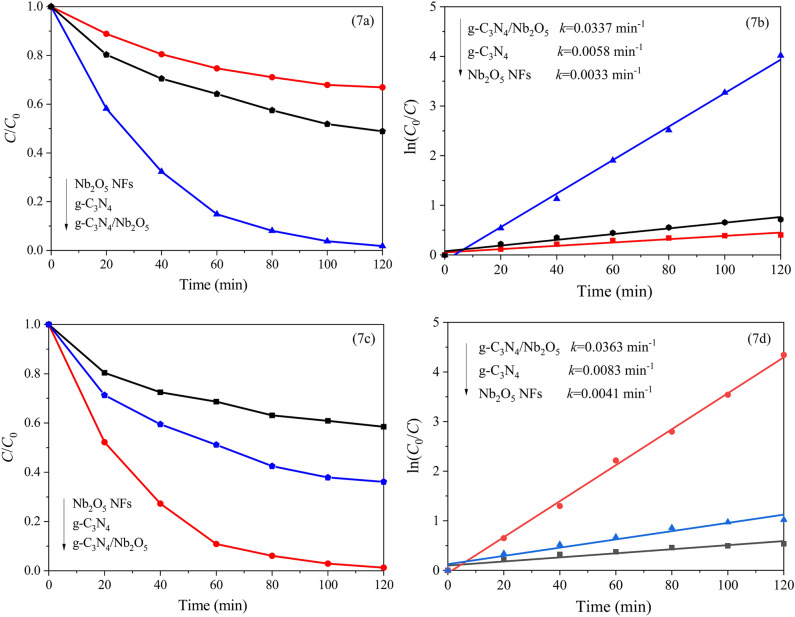
The degradation efficiency of (**a**) RhB and (**b**) phenol, kinetics curves for (**c**) RhB and (**d**) phenol over g-C_3_N_4_, Nb_2_O_5_ NFs and g-C_3_N_4_/Nb_2_O_5_ heterojunction under visible light irradiation.

The photocatalytic activities of the synthetized samples were investigated by decomposing rhodamine B (RhB) under visible light irradiation. As shown in Fig. 7a, compared to Nb_2_O_5_ NFs and g-C_3_N_4_, g-C_3_N_4_/Nb_2_O_5_ heterojunction possessed the highest photocatalytic degradation rate owing to the existence of synergistic effect between g-C_3_N_4_ and Nb_2_O_5_. After 120 min irradiation, the photodegradation efficiency of RhB for g-C_3_N_4_/Nb_2_O_5_ heterojunction was 98.1%. The superior photocatalytic activity of g-C_3_N_4_/Nb_2_O_5_ heterojunction over RhB could also be confirmed from kinetics experiment. On the base of the rate equation of − ln(*C*/*C*_0_) = *kt*, *k* representing the degradation rate constant, the kinetic curves for the RhB photodegradation over three photocatalysts were obtained, as shown in Fig. 7b. It was obviously that the *k* value of g-C_3_N_4_/Nb_2_O_5_ heterojunction is larger than that of Nb_2_O_5_ NFs and g-C_3_N_4_. In addition, phenol, a colorless organic pollutant model, was selected to further evaluate the photocatalytic activity of g-C_3_N_4_/Nb_2_O_5_ heterojunction. It can be found that the g-C_3_N_4_/Nb_2_O_5_ heterojunction also displayed the highest photocatalytic activity for phenol degradation. It was observed that essentially complete degradation of phenol occured over 120 min for g-C_3_N_4_/Nb_2_O_5_ heterojunction (Fig. 7c). The degradation rate constant of phenol over different catalysts were also calculated by the above rate equation. It is found that g-C_3_N_4_/Nb_2_O_5_ heterojunction still achieved the highest apparent rate constant among all these samples (Fig. 7d). Meanwhile, the total organic carbon (TOC) removal rate of g-C_3_N_4_/Nb_2_O_5_ heterojunction sample for degradation of RhB and phenol were shown in Fig. S4. It can be seen that the TOC of both solutions decreased as the reaction time increased and reached almost to zero indicating that mineralization of the pollutants occur during the photocatalytic reaction^64^. Table 1 presented the photocatalytic degradation rate of various g-C_3_N_4_/Nb_2_O_5_ composites, which are comparable to as-synthesized g-C_3_N_4_/Nb_2_O_5_ heterojunction, is exhibited remarkable degradation rate.

**Table 1:** Summary of the photocatalytic activity on various g-C_3_N_4_/Nb_2_O_5_ composites.

Preparation method	Morphology of Nb_2_O_5_	Organic pollutants	Initial concentration (mg L^−1^)	Removal (%)	Conditions	Ref
Calcination	Nanoparticles	Tetracycline hydrochloride	20	76.2	Visible light, 150 min	^40^
				90.1	Simulated solar light, 60 min	
Sonochemical method	Nanoparticles	Amiloride	10	89	Visible light, 180 min	^39^
		Rhodamine B	10	81	Visible light, 90 min	
Thermal oxidation and hydrothermal treatment	Nanoparticles	Methylene blue	10	82	UV light, 210 min	^3,8^
			10	65	Visible light, 210 min	
Electrospinning	Nanofibers	Rhodamine B	10	98.1	Visible light, 120 min	Present work
		Phenol	10	98.7	Visible light, 120 min	

### Photocatalysis mechanism

**Figure 8:**
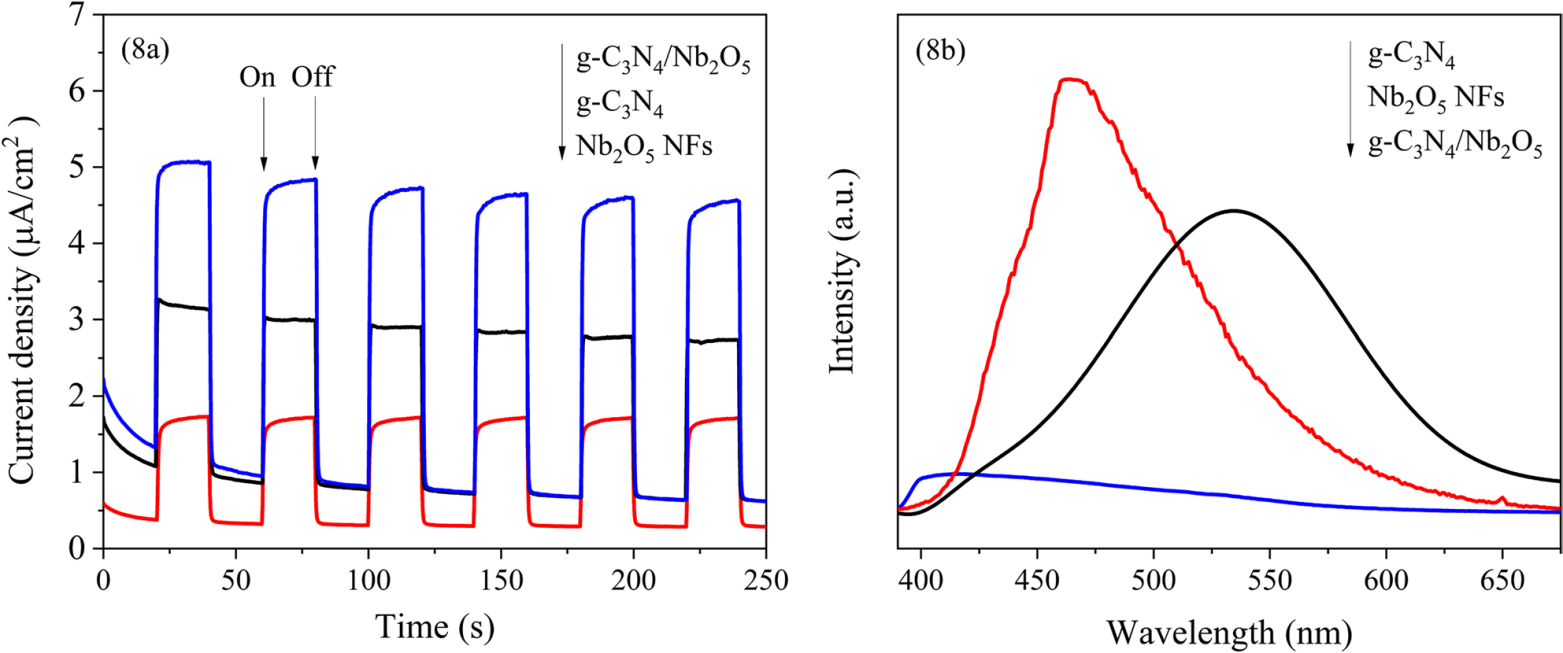
(**a**) Transient photocurrent response and (**b**) PL spectra of g-C_3_N_4_, Nb_2_O_5_ NFs and g-C_3_N_4_/Nb_2_O_5_ heterojunction.

Investigating the lifetime of photoexcited charge carriers is fundamental to understand the photocatalysis mechanism. The photocurrent responses of g-C_3_N_4_, Nb_2_O_5_ NFs and g-C_3_N_4_/Nb_2_O_5_ heterojunction were undertaken to manifest the separation efficiency of the electron–hole pairs. As shown in Fig. 8a, g-C_3_N_4_/Nb_2_O_5_ heterojunction exhibited the strongest photocurrent density in comparison with that of the single component, which reflected the reduced interface resistance and the enhanced separation and migration efficiency of the photoinduced electron–hole pairs in g-C_3_N_4_/Nb_2_O_5_ heterojunction^65^. This was also consistent with the photoluminescence (PL) spectroscopy results. Based on Fig. 8b, it can be seen that the peak intensities gradually decrease in the order of g-C_3_N_4_, Nb_2_O_5_ NFs and g-C_3_N_4_/Nb_2_O_5_ heterojunction, indicating the g-C_3_N_4_/Nb_2_O_5_ heterojunction possessing the lowest electron–hole recombination efficiency. It can be concluded that the recombination of photogenerated charge carriers were inhibited effectively due to the heterojunction construction of g-C_3_N_4_ and Nb_2_O_5_^66^.

**Figure 9:**
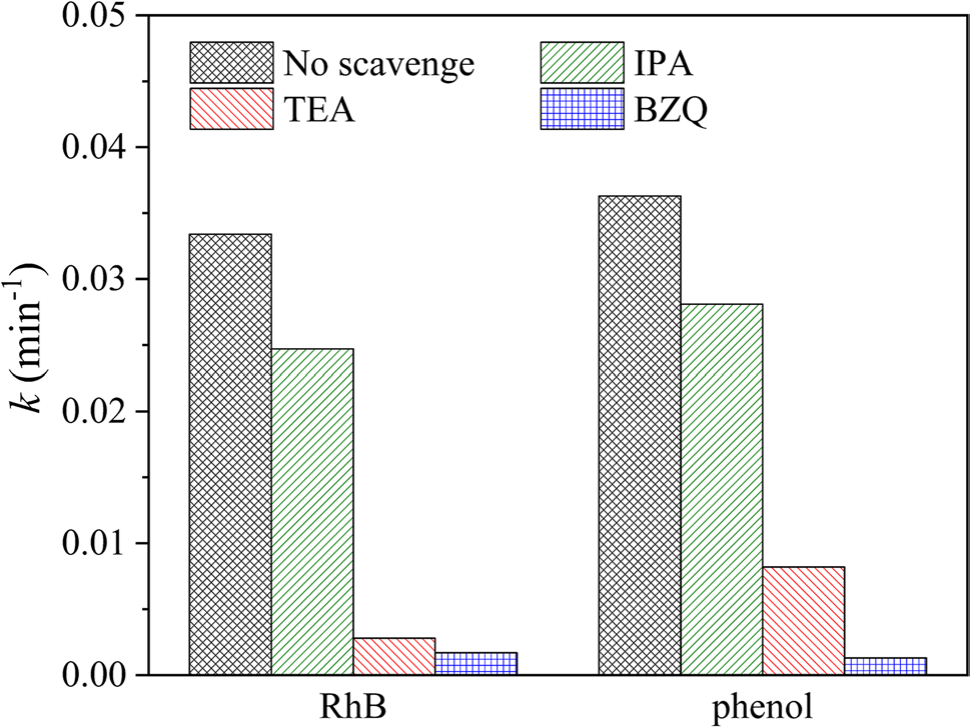
Effects of different scavengers on RhB and phenol photodegradation over the g-C_3_N_4_/Nb_2_O_5_ heterojunction.

On the other side, hydroxyl radical (·OH), hole (h^+^) and superoxide radical anion (·O_2_^–^) as major active species play key roles in photocatalytic reaction. To elucidate the mechanism of g-C_3_N_4_/Nb_2_O_5_ heterojunction, free radicals trapping experiments were performed to confirm the active species contributing to RhB and phenol photodegradation. In these experiments, isopropanol (IPA), triethanolamine (TEA) and p-benzoquinone (BZQ) acted as the scavenger for ·OH, h^+^ and ·O_2_^–^, respectively. Figure 9 revealed the photocatalytic efficiencies of RhB and phenol with g-C_3_N_4_/Nb_2_O_5_ heterojunction after adding various scavengers. The reaction rate constant showed almost unchanged in the presence of IPA, while the addition of TEA and BZQ led to an obviously decrease of the reaction rate constant. In view of the above results, it can be proposed that ·O_2_^–^ is the main active species and h^+^ also play important role in g-C_3_N_4_/Nb_2_O_5_ heterojunction for RhB and phenol degradation.

**Figure 10:**
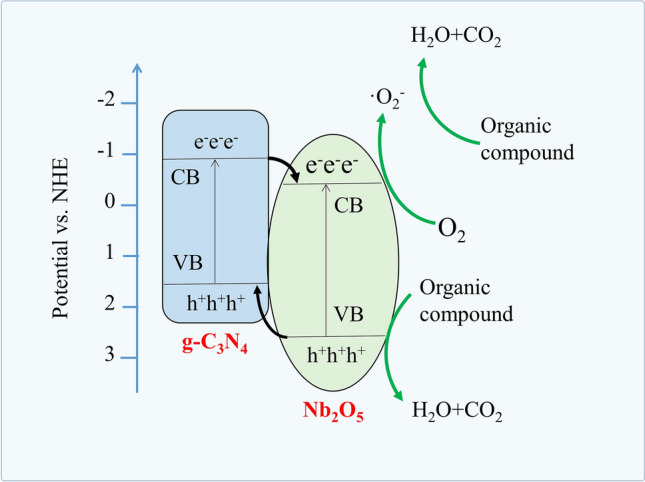
Schematic diagram of photogenerated charges in g-C_3_N_4_/Nb_2_O_5_ heterojunction.

Based on the above analysis and experiments, the mechanism was proposed and schematically illustrated in the Fig. 10. It was well understood that the distinction of CB edge potentials between semiconductors strongly promoted the electrons transfer at the heterojunction interface and reduced the recombination of carriers, ultimately improved the photodegradation activity of heterojunction^67^. When g-C_3_N_4_/Nb_2_O_5_ heterojunction was exposed to irradiation, g-C_3_N_4_ component was easy to be excited by visible light due to its narrow band gap. And the photogenerated electrons were easy to transfer from CB of g-C_3_N_4_ to that of Nb_2_O_5_ because the CB edge potential of g-C_3_N_4_ (− 0.98 eV) was more negative than that of Nb_2_O_5_ (− 0.59 eV). The photogenerated electrons could react with dissolved O_2_ near the Nb_2_O_5_ NFs to form the main active specie of ·O_2_^–^, which was mostly responsible for degrading pollutants. Similarly, the VB edge potential of g-C_3_N_4_ (1.72 eV) was also more negative than that of Nb_2_O_5_ (2.61 eV), and the photogenerated h^+^ transfer to VB of g-C_3_N_4_ from VB of Nb_2_O_5_ through the tight heterojunction interface. The residual h^+^ on VB of Nb_2_O_5_ could oxidize organic molecules to photodegradation products directly. Moreover, the multi-point connected Nb_2_O_5_ nanofiber and porous g-C_3_N_4_ were also helpful to promote the electron migration.

### Reusability of g-C_3_N_4_/Nb_2_O_5_ heterojunction

**Figure 11:**
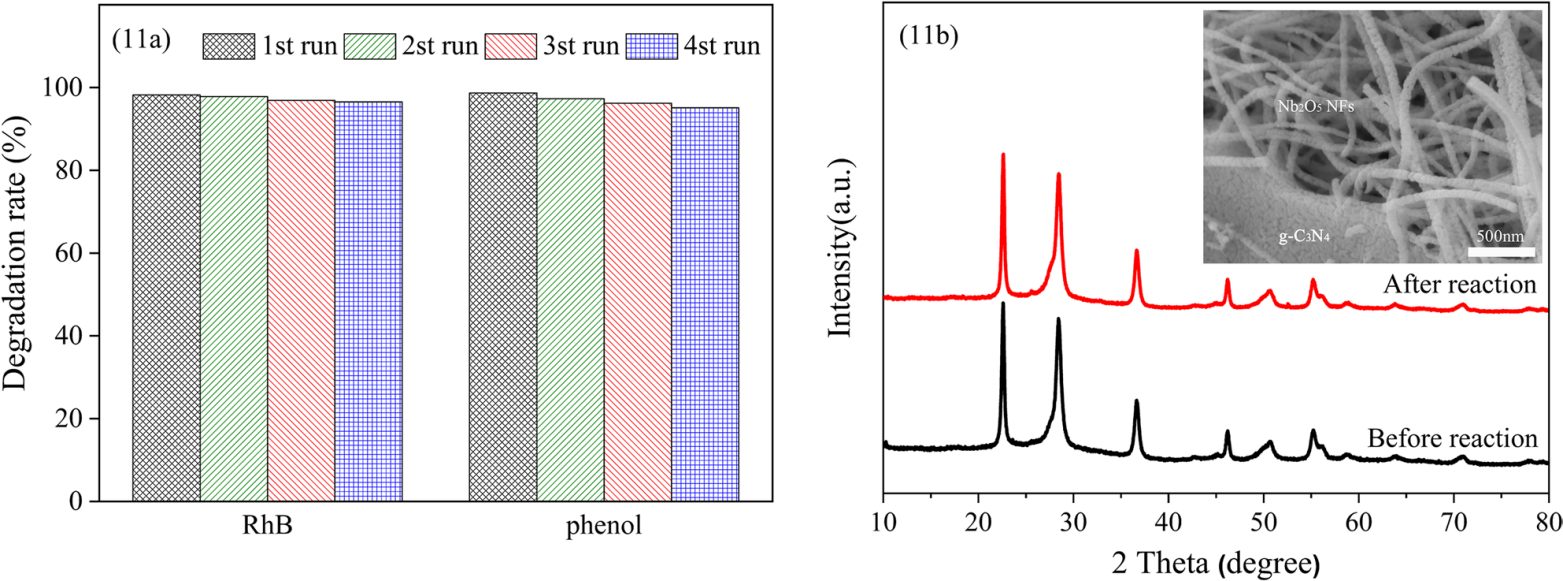
(**a**) The reusability of g-C_3_N_4_/Nb_2_O_5_ heterojunction: photodegradation of RhB and phenol. (**b**) XRD patterns of g-C_3_N_4_/Nb_2_O_5_ heterojunction before and after photodegradation, and a typical SEM image (inset) of g-C_3_N_4_/Nb_2_O_5_ heterojunction after the photodegradation.

To investigate the stability and reusability of the g-C_3_N_4_/Nb_2_O_5_ heterojunction, recycling test of the photodegradation were performed. After each photodegradation cycle, the catalyst was filtered from the solution and dried at 80 °C for further use. As depicted in Fig. 11a, the g-C_3_N_4_/Nb_2_O_5_ heterojunction showed no obvious reduction of photocatalytic activity after four cycles for both RhB and phenol, demonstrating its excellent stability and reusability for the multiple times for organic pollutants degradation. And the XRD patterns of the g-C_3_N_4_/Nb_2_O_5_ heterojunction before and after the reaction were similar (Fig. 11b). From a typical SEM image of g-C_3_N_4_/Nb_2_O_5_ heterojunction (inset of Fig. 11b) after photocatalytic reaction, it can be clearly seen that the Nb_2_O_5_ NFs is still tightly attached with g-C_3_N_4_ nanosheet, which indicates that the morphology of g-C_3_N_4_/Nb_2_O_5_ heterojunction remains unchanged after photocatalytic reaction.

## Conclusions

In summary, the g-C_3_N_4_/Nb_2_O_5_ heterojunction was successfully synthesized using a simple and facile electrospinning-calcination process and displayed excellent photocatalytic activity for RhB and phenol degradation. Particularly, the as-prepared g-C_3_N_4_/Nb_2_O_5_ heterojunction exhibited higher photocatalytic activities towards the photodegradation of RhB and phenol under visible irradiation, compared to the pure g-C_3_N_4_ and Nb_2_O_5_ phases. The low bandgap energy (2.84 eV) as well as the synergistic effect between Nb_2_O_5_ NFs and g-C_3_N_4_ in the g-C_3_N_4_/Nb_2_O_5_ heterojunction enhanced the photocatalytic activity, which were beneficial to a rapid photoinduced charge separation in the electron transfer process and a slow charge recombination. In addition, both superoxide radical anion (·O_2_^–^) and hole (h^+^) were the major oxidative species for RhB and phenol degradation over the g-C_3_N_4_/Nb_2_O_5_ heterojunction photocatalyst. This work may provide a promising future of applying photocatalyst to solving dye pollutant problems and solar energy conversion.

## Supplementary Information

Supplementary Information.
