# Supplementary material for: Electrospinning preparation of g-C3N4/Nb2O5 nanofibers heterojunction for enhanced photocatalytic degradation of organic pollutants in water
Source: Sci Rep. 2021 Nov 25;11:22950. doi: 10.1038/s41598-021-02161-x (PMC8617280; doi:10.1038/s41598-021-02161-x)
Supplement: Supplementary file 1 — Supplementary Information. [file 41598_2021_2161_MOESM1_ESM.docx]

**Supporting Information**

Electrospinning preparation of g-C_3_N_4_/Nb_2_O_5_ nanofibers heterojunction for enhanced photocatalytic degradation of organic pollutants in water

Lu Wang^1,2^, Ya Li^3^, and Pingfang Han^1,^*

^1^ College of Biotechnology and Pharmaceutical Engineering, Nanjing Tech University, Nanjing 211816, China

^2^ Nantong Vocational University, Nantong 226007, China

^3^ Nantong College of Science and Technology, Nantong 226007, China

*Corresponding authors: Pingfang Han, hpf@njtech.edu.cn

**Material characterizations**

Scanning electron microscopy (SEM, Hitachi SU8010) and transmission electron microscope (TEM, JEOL JEM-2100F) were taken to characterize the morphologies and structures of the samples. X-ray diffraction (XRD, Rigaku ULTIMA IV diffractometer) was used to determine the crystal structure with Cu Kα radiation (1.5404 Å) in the range 10-80° (2θ). Fourier transform infrared (FTIR) spectra were measured with a Bruker Tensor 27 instrument. Thermogravimetric analysis (TGA) was carried out using a Perkin EImer Simultaneous Thermal Analyzer (STA 8000, America) at a heating rate of 10 °C/min. X-ray photoelectron spectroscopy (XPS, Thermo Scientific K-ALPHA) was taken to test the surface composition of the samples with Al Kα radiation. Nitrogen adsorption-desorption isotherm measurements were performed on a Micromeritics ASAP 2460 to analyze the textural properties of the samples. The UV-Vis diffused reflectance spectra were obtained with a Shimadzu UV-2600 spectrophotometer using polytetrafluoroethylene as the background. Photoluminescence (PL) spectra were recorded using a Hitachi F-4600 spectrophotometer with an excitation wavelength of 325 nm. The photocurrent responses were implemented on a Shanghai Chenhua 760 E electrochemical system by a standard three-electrode system.

**Photocatalytic test**

The photocatalystic activity was tested by degrading Rhodamine B (RhB) (or phenol) under visible light of 300-W Xenon lamp (PerkinElmer Co.) with a UV cutoff filter (λ greater than 400 nm). Typically, 50 mL of RhB (or phenol) aqueous solution (10 mg/L) containing 50 mg of photocatalyst was stirred in dark for 30 min for absorption-desorption equilibrium. Afterwards, the xenon lamp was turned on and 3 mL of suspension solution was taken out at a fixed interval of time. Subsequently, the absorbance of the RhB (or phenol) solution was monitored using Shanghai Mapada UV-1800 spectrometer at the maximum absorption wavelength after removing the insolubles. Total organic content (TOC) was analyzed on a Shimadzu TOC-V CPH TOC analyzer.

**
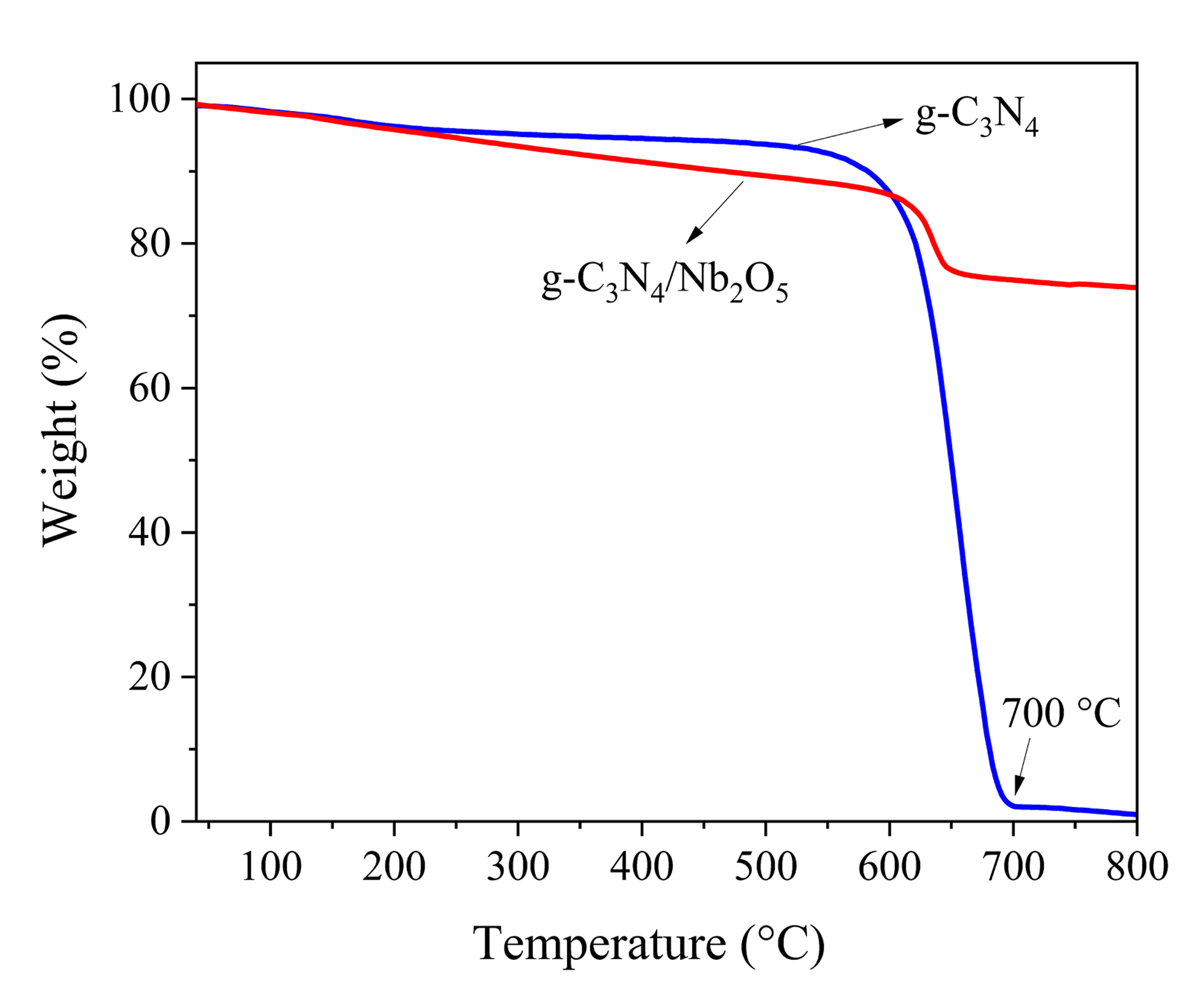
**

**Figure S1.** TGA curves of g-C_3_N_4_ and g-C_3_N_4_/Nb_2_O_5_ heterojunction.


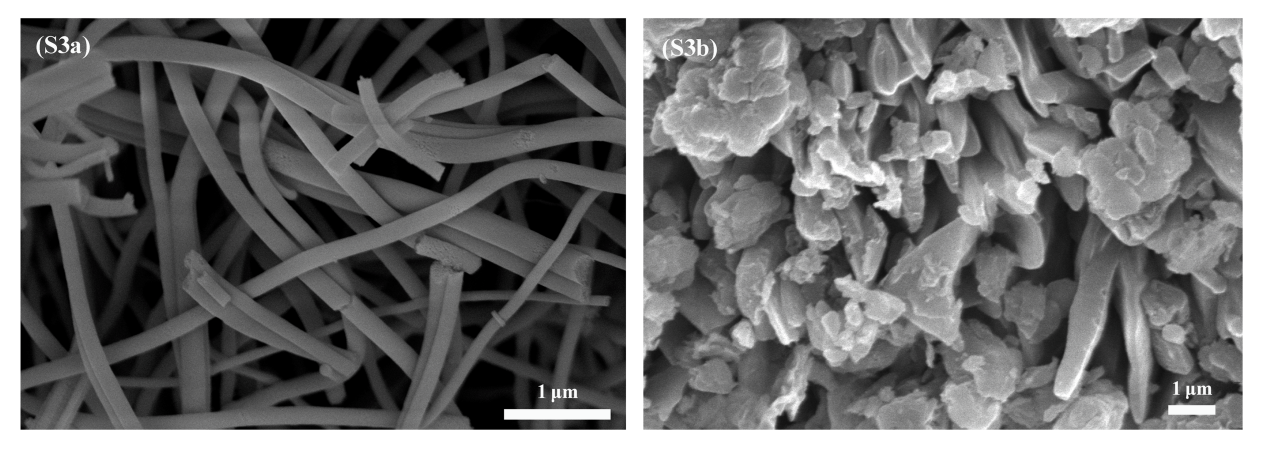


**Figure S2.** SEM images of (a) Nb_2_O_5_ NFs and (b) g-C_3_N_4_.

**
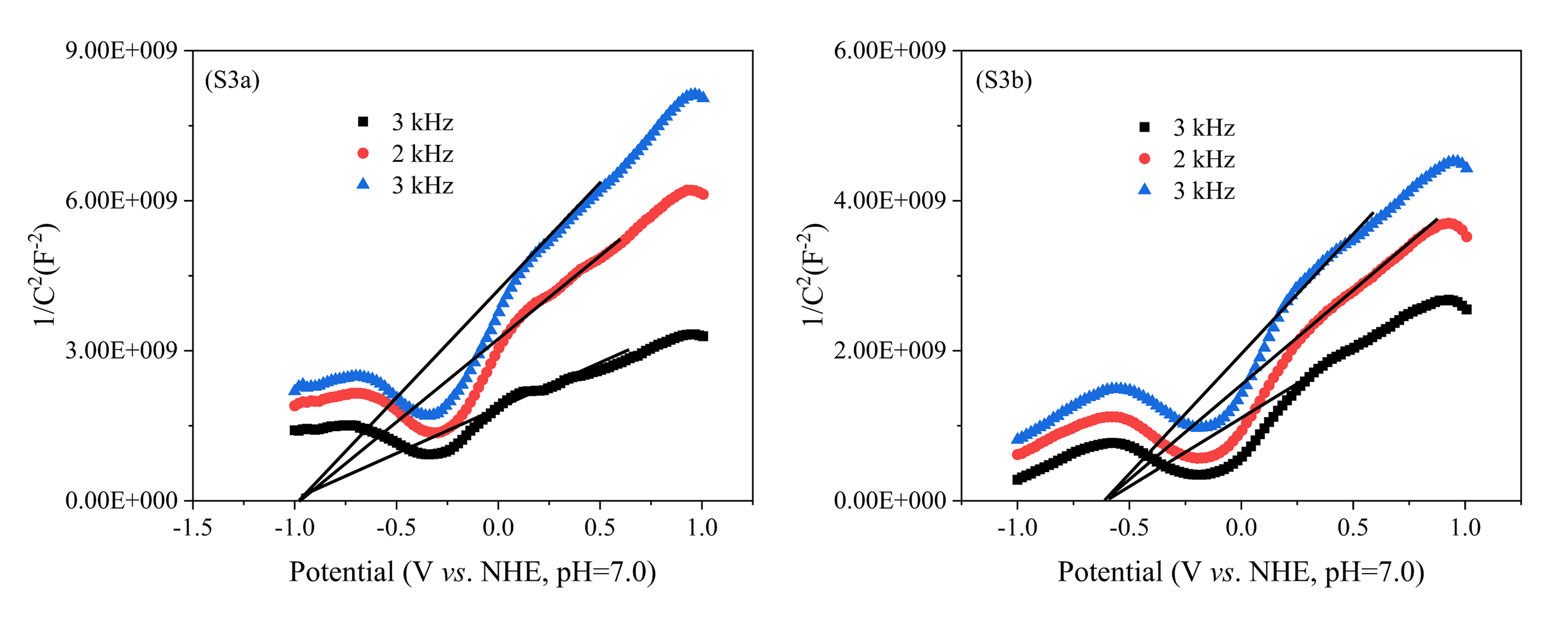
**

**Figure S3.** Mott-Schottky plots of (a) g-C_3_N_4_ and (b) Nb_2_O_5_ NFs.


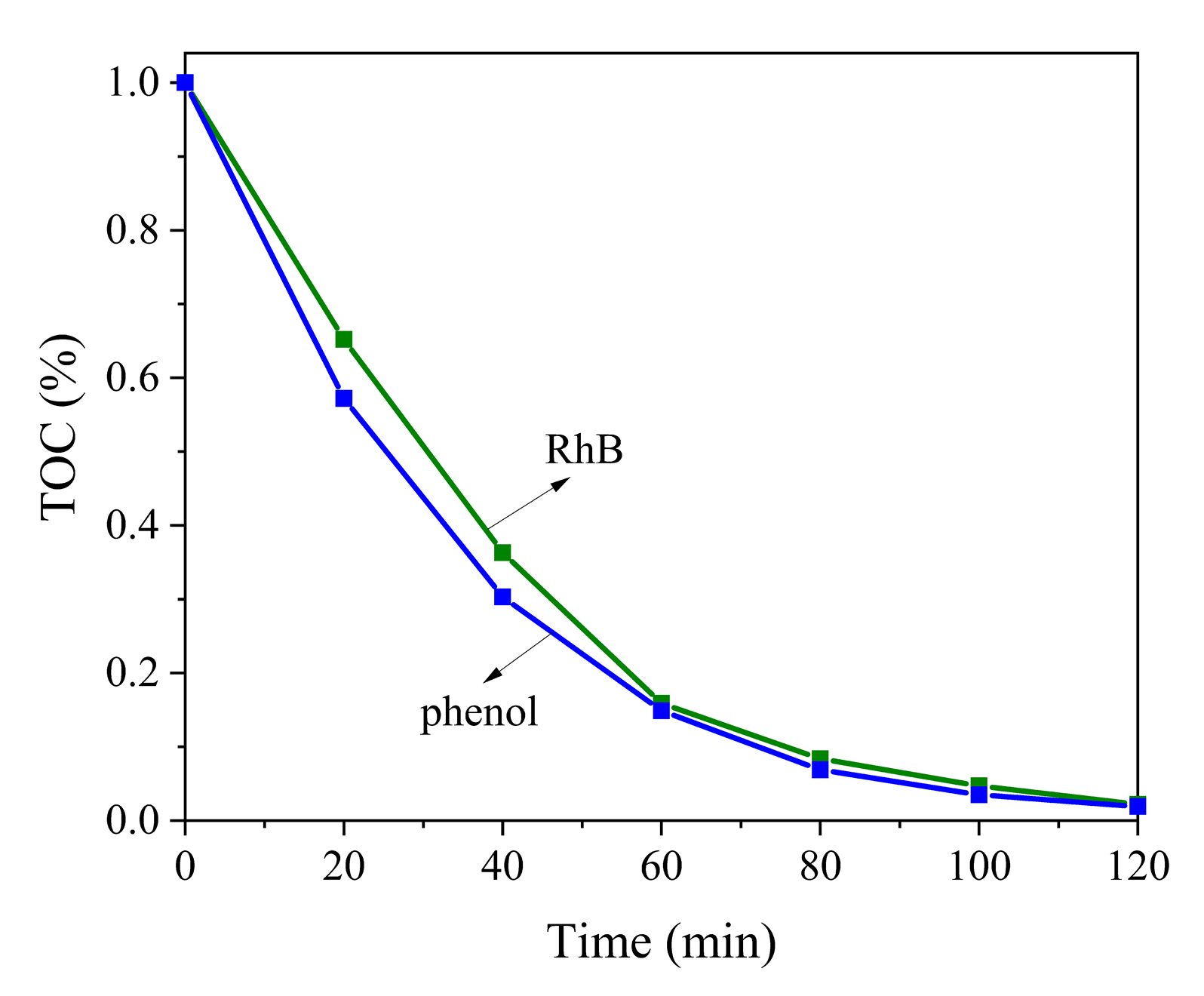


**Figure S4.** TOC removal efficiency of RhB and phenol using g-C_3_N_4_/Nb_2_O_5_ as photocatalyst under visible light irradiation.
